# Identification of multiple functional receptors for tyramine on an insect secretory epithelium

**DOI:** 10.1038/s41598-017-00120-z

**Published:** 2017-03-13

**Authors:** Haiying Zhang, Edward M. Blumenthal

**Affiliations:** Department of Biological Sciences, Marquette University, P.O. Box 1881, Milwaukee, WI 53201-1881 USA

## Abstract

The biogenic amine tyramine (TA) regulates many aspects of invertebrate physiology and development. Although three TA receptor subtypes have been identified (TAR1-3), specific receptors have not been linked to physiological responses in native tissue. In the Malpighian (renal) tubule of *Drosophila melanogaster*, TA activates a transepithelial chloride conductance, resulting in diuresis and depolarization of the transepithelial potential. In the current work, mutation or RNAi-mediated knockdown in the stellate cells of the tubule of *TAR2* (*tyrR*, *CG7431*) resulted in a dramatic reduction, but not elimination, of the TA-mediated depolarization. Mutation or knockdown of *TAR3* (*tyrRII*, *CG16766*) had no effect. However, deletion of both genes, or knockdown of *TAR3* on a *TAR2* mutant background, eliminated the TA responses. Thus while TAR2 is responsible for the majority of the TA sensitivity of the tubule, TAR3 also contributes to the response. Knockdown or mutation of *TAR2* also eliminated the response of tubules to the related amine octopamine (OA), indicating that OA can activate TAR2. This finding contrasts to reports that heterologously expressed TAR2 is highly selective for TA over OA. This is the first report of TA receptor function in a native tissue and indicates unexpected complexity in the physiology of the Malpighian tubule.

## Introduction

The biogenic amine tyramine (TA) is an important but relatively understudied modulator of many aspects of invertebrate physiology. Long considered to be simply an intermediate in the synthesis of octopamine (OA) from tyrosine, TA is now recognized to have physiological effects independent of OA^1, 2^. These include effects on egg melanisation^3^, sex pheromone production^4^, olfactory behavior^5^, locomotion and flight^6–9^, neuromuscular transmission^5, 10^, sleep^11^, appetite^12^, behavioral responses to cocaine and ethanol^13–15^, and muscular contractions^16–18^. Putative tyraminergic neurons, which are immunoreactive against TA but not OA, have been identified in both *Drosophila* and locust^10, 16, 19, 20^. In the nematode *C. elegans*, a clear role for TA in modulating the escape response has been demonstrated^21–24^. Interestingly, TA is also one of the “trace amines” found in vertebrates and has been linked to human disorders such as migraines and ADHD^25–27^.

Multiple types of insect G-protein coupled receptors are activated by TA; these receptors have been classified into three groups of OA receptors and three groups of TA receptors according to a system proposed by Evans and Maqueira^28^ and subsequently modified^29–31^. TA can act as an agonist of many OA receptor subtypes, but the OA receptors show varying degrees of selectivity for OA over TA^31–37^. The first subtype of TA receptor, the “Oct-TyrR” receptor class, recently renamed TAR1 receptors^29^, is activated with a weak selectivity for TA over OA^38–44^. Receptors in this class inhibit adenylyl cyclase, although in some systems they can also trigger calcium release^45^. Members of a second class of TA receptors, the TAR2 receptors, are coupled to calcium release and are reported to be extremely selective for TA. When expressed in mammalian 293 or CHO cells, the TAR2 receptors from *Drosophila*, the silk moth *Bombyx mori*, and the rice stem borer *Chilo suppressalis* are activated by low nanomolar concentrations of TA but are completely insensitive to micromolar doses of OA^29, 46–48^. A third tyramine receptor, TAR3, is also present in the *Drosophila* genome; expression of *Drosophila melanogaster* TAR3 in CHO cells results in a receptor that couples both to calcium release and to the inhibition of adenylyl cyclase and that is moderately selective for TA over OA and other amines^29^.

The Malpighian tubules (MTs) of *Drosophila melanogaster* are the best characterized system for studying TA signaling in a native insect tissue. These epithelial tubes produce primary urine through the transport of water and solutes from the surrounding hemolymph^49^. Secretion of primary urine by the MTs is driven by the active transport of cations across the apical membrane of the primary cell type, the principal cells. Upon application of nanomolar concentrations of TA or of the peptide leucokinin, primary urine production is stimulated and the transepithelial potential (TEP) depolarized due to an increase in transepithelial chloride conductance^50, 51^. This increase in chloride conductance is associated with an increase in calcium levels in a secondary cell type, the stellate cell, and is dependent upon the expression of a specific chloride channel in the stellate cells^52–54^. MTs also show a depolarizing response to OA and dopamine, but only at concentrations several orders of magnitude higher than TA^51^. Finally, we have shown that TA can be synthesized from tyrosine by tyrosine decarboxylase expressed by the *Tdc1* gene in the principal cells, suggesting that TA acts as an agent of cell-cell communication in the MT^55^.

Despite the growing body of literature on the identification and pharmacological characterization of insect TA receptors, no studies have examined the function of specific TA receptors in their native cellular environment. In the current work, we aim to bridge the gap between the physiological effects of TA and the function of specific TA receptors. We use the power of *Drosophila* genetics and the sensitivity of the isolated MT to identify TAR2, and to a lesser extent TAR3, as responsible for the TA response in the MT. Surprisingly, the agonist profile of the *Drosophila* TAR2 is strikingly different in native tissue than in heterologous cells. Finally, we have generated mutant fly lines that will facilitate the identification of further physiological roles for specific TA receptors in *Drosophila*.

## Results

For consistency with the published classification of TA receptors, we will refer to the gene encoding the *Drosophila* type 2 TA receptor, also called *CG7431* or *TyrR*, as *TAR2*, and the gene encoding the *Drosophila* type 3 TA receptor, also called *CG16766* or *TyrRII*, as *TAR3*. According to FlyAtlas^56^, expression of both genes is enriched in the larval MT (10.8-fold for *TAR2* and 4.0-fold for *TAR3*) but not in the adult MT (1.2-fold for *TAR2* and 1.3-fold for *TAR3*). However, an earlier transcriptome analysis by the same investigators^57^ identified *TAR2* as the sixth most highly enriched receptor in the adult MT (8.5-fold enrichment). The reason for this discrepancy is unclear. Additionally, in the beetle *Nicrophorus vespilloides*, TAR2 is reported to have expression levels in the MT at least 10-fold higher than other TA or OA receptor genes^58^.

**Figure 1:**
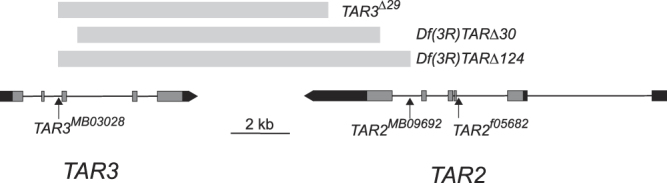
Genomic organization of the TAR2 and TAR3 genes. Boxes indicate exons, with lighter shading indicating coding regions and darker shading indicating untranslated regions. The precise sizes of the deletions created in this study are 12,084 bp (*Df*(*3R*)*TAR*Δ*124*), 10,367 bp (*Df*(*3R*)*TAR*Δ*30*), and 9,294 bp (*TAR3* ^Δ*29*^).

**Figure 2:**
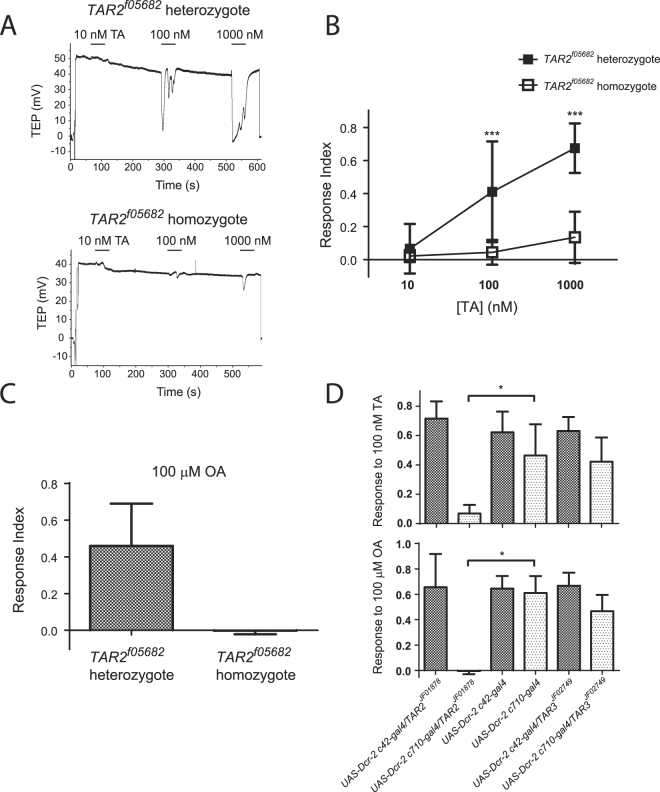
(**A**) Representative responses to 10, 100, and 1000 nM TA of a *TAR2* ^*f05682*^ heterozygote (upper trace) and homozygote (lower trace). (**B**) Dose-response curve for TA in *TAR2* ^*f05682*^ heterozygotes and homozygotes. ***Significant difference between genotypes, p < 0.001, 2-way ANOVA and Bonferroni post-test, n = 8–10 tubules per genotype. (**C**) Response of *TAR2* ^*f05682*^ heterozygotes and homozygotes to 100 μM OA. The average response amplitude of homozygotes does not differ from zero, 1-sample t-test, p = 0.76. n = 9 tubules per genotype. (**D**) Effect of *TAR2* and *TAR3* RNAi on responses to 100 nM TA (upper graph) and 100 μM OA (lower graph). The responses of RNAi tubules were compared to those of the appropriate parental line by Kruskal-Wallis and Dunn’s multiple comparison tests. Asterices indicate that the only difference seen was upon RNAi of *TAR2* in the stellate cells. Knockdown of TAR2 in the stellate cells did not eliminate the response to TA (1-sample t-test, p = 0.01) but did eliminate the response to OA (p = 0.82). n = 8–11 tubules per condition.

To study the role of the *Drosophila* TAR2 receptor in the TA response of the MT, we obtained a fly line carrying an allele of *TAR2*, *TAR2* ^*f05682*^, caused by a transposon insertion in the second intron of the gene (Fig. 1). Quantification of *TAR2* expression in the MT by real-time RT-PCR indicated a dramatic reduction in transcript levels compared to heterozygous siblings. Only 1 of 3 homozygous cDNA samples gave a positive qPCR signal for *TAR2*. Based on the quantification of that signal and comparison to a dilution series of heterozygous cDNA, we estimate that *TAR2* transcript abundance was reduced by 200–1000 fold in the homozygous mutants (data not shown). Electrophysiological recording of the TA response in isolated MTs showed a significant reduction, but not an elimination, of TA sensitivity in homozygotes compared to heterozygotes. Figure 2A shows representative responses to 10, 100, and 1000 nM TA; a clear response to the highest dose was observed in the homozygote. A dose-response curve to TA demonstrated significant reductions in the average response to 100 nM and 1000 nM TA in homozygotes (p < 0.001, 2-way ANOVA and Bonferroni posthoc test) but an average response to 1000 nM TA in the homozygotes that differed significantly from zero (p = 0.005, 1-sample t-test) (Fig. 2B). Sensitivity to 100 μM OA was completely abolished in the homozygotes (Fig. 2C). In contrast, there was no difference in the response to the peptide drosokinin (100 pM), the *Drosophila* ortholog of leucokinin, between heterozygotes and homozygotes (Fig. S1). Flies homozygous for another insertion in the same intron of *TAR2*, *TAR2* ^*LL06812*^, showed a less dramatic reduction in the TA responses of isolated MTs (data not shown).

We used cell-type specific knockdown of *TAR2* expression to determine its site of functional expression in the MT. Driving the inducible RNAi transgene *TAR2* ^*JF01878*^ with the principal cell-specific driver *c42-gal4* resulted in responses to 100 nM TA and 100 μM OA that were unchanged compared with parental controls. In contrast, expression of the RNAi transgene in the stellate cells with the *c710-gal4* driver caused a dramatic reduction in the response to 100 nM TA and an elimination of the response to 100 μM OA (Fig. 2D). To determine the extent of the knockdown in these flies, we measured *TAR2* transcript levels in the MT by quantitative RT-PCR. Surprisingly, *TAR2* expression in the c710 knockdown flies was not reduced relative to the c42 knockdowns or parental controls (Fig. S2).

Neither knockdown nor mutation of *TAR2* resulted in the elimination of responses to high concentrations of TA. To test the possibility that the homologous receptor TAR3 might also function in the MTs, we knocked down *TAR3* expression with the RNAi transgene *TAR3* ^*JF02749*^. Tubules in which *TAR3* was knocked down in either the principal cells or the stellate cells displayed responses to 100 nM TA and 100 μM OA that were unchanged compared with those of parental controls (Fig. 2D). We were unable to determine the extent of *TAR3* knockdown in these MTs by quantitative RT-PCR due to the extremely low levels of *TAR3* expression in the MTs (data not shown).

The interpretation of the experiments described above is limited by the fact that neither the available *TAR2* mutants nor the RNAi-mediated knockdown of *TAR2* or *TAR3* completely abolishes expression of the target gene. To generate complete null mutations of both *TAR2* and *TAR3*, we used transposase-mediated recombination between Minos transposon insertions in the two adjacent genes. Three viable deletion lines were generated and the deletions were mapped by PCR and sequencing (see methods). In two of these deletions, *Df*(*3R*)*TAR*Δ*30* and *Df*(*3R*)*TAR*Δ*124*, significant portions of the coding sequences of both *TAR2* and *TAR3* are deleted (Fig. 1), and we consider both of these deletions to be null for both TAR genes. In the third line, *TAR3* ^Δ*29*^, the majority of the *TAR3* coding sequence is deleted along with the final 758 bp of the predicted 1984 bp *TAR2* 3′ untranslated region. Quantitative RT-PCR indicated no difference in *TAR2* transcript abundance in the MTs of *TAR3* ^Δ*29*^ homozygotes compared with heterozygotes (data not shown), and so we consider this deletion to be a specific null mutation of *TAR3*. The deletions in the three lines did not knock out any other genes, as the breakpoints of all deletions were contained within the adjacent *TAR2* and *TAR3* genes.

**Figure 3:**
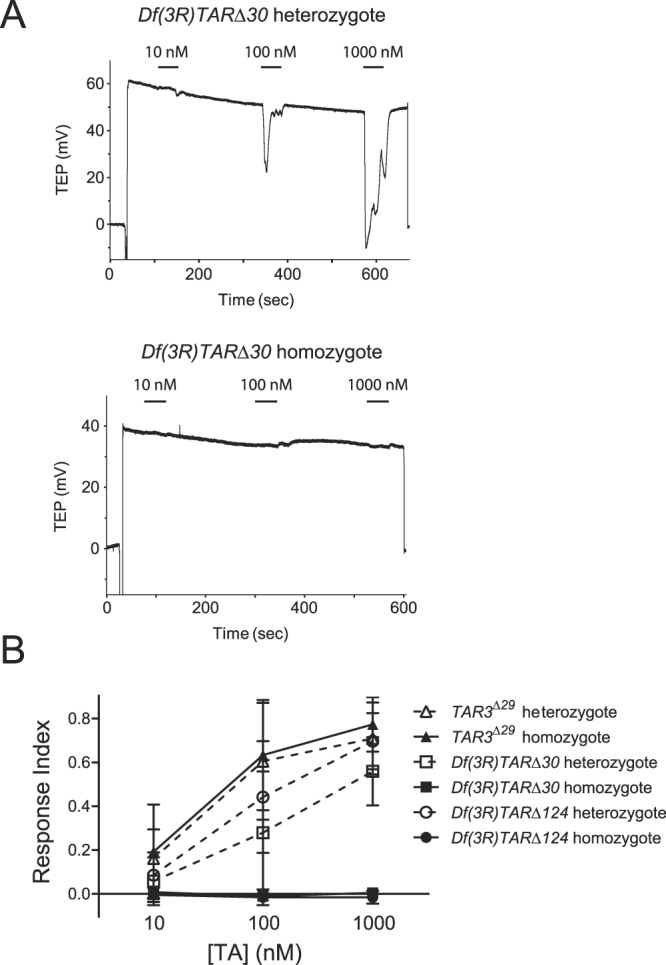
Response of TAR deletion mutants to TA. (**A**) Representative traces showing the response to 1000 nM TA of a *Df*(*3R*)*TAR*Δ*30* heterozygote (upper trace) and homozygote (lower trace). (**B**) Dose-response curve to TA for *TAR3* ^Δ*29*^, *Df*(*3R*)*TAR*Δ*30*, and *Df*(*3R*)*TAR*Δ*124* heteroyzygotes and homozygotes. The mean responses of *Df*(*3R*)*TAR*Δ*30*, and *Df*(*3R*)*TAR*Δ*124* homozygotes to each concentration of TA were either not significantly different from zero by a 1-sample test or were negative. There were no differences in the responses of *TAR3* ^Δ*29*^ homozygotes vs heterozygotes, p > 0.05 by 2-way ANOVA and Bonferroni post-test. n = 7–12 tubules per point.

Tubules isolated from homozygotes of either *TAR2-TAR3* deletion line were completely unresponsive to TA concentrations up to 1000 nM and to 100 μM OA (Figs 3, S3). The deletion of these two TAR genes did not abolish the ability of tubules to respond to depolarizing stimuli, as there was no decrement in the response to 100 pM drosokinin (Fig. S3). In contrast, deletion of only *TAR3* had no effect on the amplitude of responses to either TA or OA (Figs 3, S3).

**Figure 4:**
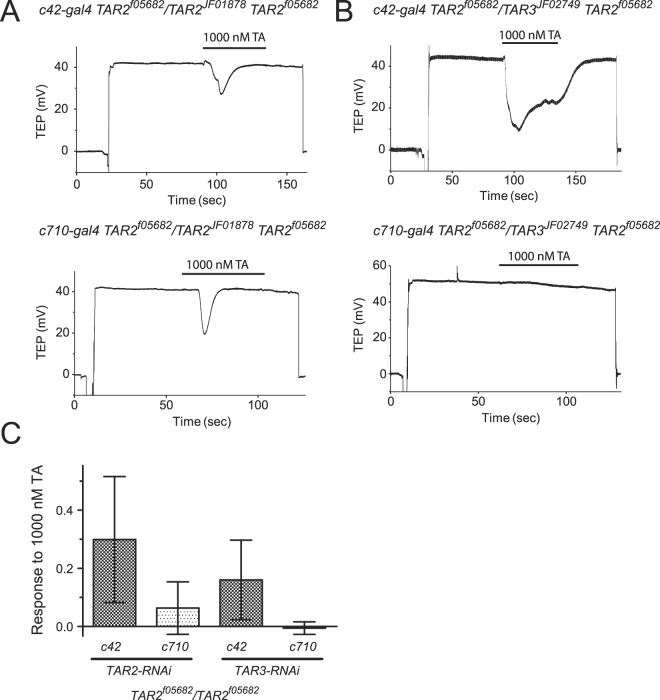
Effect of RNAi against TAR2 and TAR3 on a TAR2 mutant background. (**A**) Representative responses to 1000 nM TA from *c42-gal4 TAR2* ^*f05682*^ */TAR2* ^*JF01878*^ *TAR2* ^*f05682*^ (upper trace) and *c710-gal4 TAR2* ^*f05682*^ */TAR2* ^*JF01878*^ *TAR2* ^*f05682*^ (lower trace) tubules. (**B**) Representative responses to 1000 nM TA from *c42-gal4 TAR2* ^*f05682*^ */TAR3* ^*JF02749*^ *TAR2* ^*f05682*^ (upper trace) and *c710-gal4 TAR2* ^*f05682*^ */TAR3* ^*JF02749*^ *TAR2* ^*f05682*^ (lower trace) tubules. (**C**) Mean responses to 1000 nM of the four genotypes shown in (**A** and **B**). Knockdown of both TAR2 and TAR3 in the stellate cells caused a significant reduction in the response compared to knockdown in principal cells, p < 0.05, 1-way ANOVA and Sidak’s multiple comparisons test, but only knockdown of TAR3 in the stellate cells resulted in a mean response that was equal to zero, p = 0.45, 1-sample t-test. n = 8–9 tubules per genotype.

Taken as a whole, the data presented thus far neither confirm nor exclude a functional role for *TAR3* in the response to high concentrations of TA. To determine whether the residual response to TA observed in *TAR2* ^*f05682*^ homozygotes was due to functional expression of *TAR3*, we performed RNAi-mediated knockdown of *TAR2* or *TAR3* expression on a *TAR2* ^*f05682*^ mutant background. As shown in Fig. 4, additional knockdown of *TAR2* in the stellate cells of mutant flies reduced, but did not eliminate, the residual response to 1000 nM TA. In contrast, knockdown of *TAR3* in the stellate cells of mutant flies completely eliminated the residual TA response, indicating a minor but measurable function for the TAR3 receptor in the MT.

## Discussion

In this paper, we report the first functional characterization of individual insect TA receptor subtypes in a native tissue. Our data present clear evidence that the primary receptor for TA in the Malpighian tubules is encoded by *TAR2*, as both mutation of the gene and RNAi result in a substantial reduction of the depolarizing response to TA. At the same time it is also clear that neither the *TAR2* mutation nor RNAi wholly eliminates the TA response. In contrast, chromosomal deletions that remove both *TAR2* and *TAR3* do eliminate the TA response. There are two interpretations of these data. First, it is possible that the TAR2 receptor solely mediates the TA response, and that the small TA responses in mutants or after RNAi results from residual expression of *TAR2*. This interpretation is consistent with our detection of very low levels of properly spliced *TAR2* transcript in *TAR2* ^*f05682*^ homozygotes. The second interpretation is that both TAR2 and TAR3 contribute to the TA response, and that the residual TA response seen in *TAR2* mutants or after *TAR2* RNAi is mediated by TAR3. We believe that the results of our final experiment, in which RNAi against *TAR3* but not *TAR2* eliminates the residual TA response in *TAR2* ^*f05682*^ homozygotes, are consistent only with the second interpretation. For this final result to fit with a single functional TA receptor, one would have to argue that the *TAR3* RNAi transgene was more effective at reducing *TAR2* expression than the TAR2 RNAi transgene. Such off-target effects are theoretically possible. However, a strong off-target effect of the *TAR3* RNAi transgene is not consistent with our observation that only *TAR2* RNAi, and not *TAR3* RNAi, reduces the TA responses in otherwise wild-type tubules. Thus, we conclude that both TAR2 and TAR3 contribute to the depolarizing response of TA in the Malpighian tubule. As the TA response has been shown to be associated with an increase in intracellular calcium concentrations^54^, our data are consistent with the finding that both TAR2 and TAR3 receptors can couple to IP3 production and calcium release in heterologous expression systems^29, 48^.

Our data do reveal one important difference in the agonist profile of the TAR2 receptor in native tissue compared with heterologous cells. When expressed in mammalian cell lines, *TAR2* from several different insect species, including *Drosophila*, forms a receptor that is highly selective for TA over OA by at least five orders of magnitude^29, 46–48^. While the *Bombyx* TAR2 receptor is activated by 100 μM OA^47^, the *Drosophila* receptor is reported to be insensitive even to this high concentration^48^. In the MT however, our data show that reduction of TAR2 expression by mutation or RNAi eliminated the depolarizing response to OA, indicating that TAR2 in the tubule can be activated by OA, albeit with a potency approximately 3 orders of magnitude lower than TA. The ability of TAR2 to respond to very high micromolar concentrations of OA is unlikely to be physiologically relevant; it is however, indicative of a significant difference between the agonist selectivity of TAR2 in native tissues compared to mammalian cell lines. The explanation for this difference is unknown but perhaps could be due to abnormal processing or modification of the insect receptor by mammalian cells. We know of no other examples of the agonist profile of a GPCR differing between native tissue and a heterologous expression system.

Expression of the two TA receptors in the stellate cells, but not the principal cells, of the tubules is necessary for depolarizing responses, as evidenced by our cell-type specific RNAi data. This result is consistent with many reports demonstrating that the stellate cells are the site of action of depolarizing diuretic agents such as TA and drosokinin^50, 52–54^. Our finding that TA receptors are functionally expressed on the stellate cells is especially significant in conjunction with our previous report that TA can be synthesized *in situ* from tyrosine through the action of tyrosine decarboxylase expressed in the principal cells of the tubule^55^. Together, these two findings indicate that TA is an agent of cell-cell communication in the *Drosophila* tubule. This is the first demonstration of cell-cell communication in an insect Malpighian tubule. The functional significance of this communication is still unknown. We have previously hypothesized that significant levels of tyrosine in the hemolymph could result in constitutive activation of TA receptors on the tubule in the intact fly and that TA-mediated diuresis, because it is sensitive to hemolymph osmolality, could contribute to organismal osmoregulation^59^.

While we found that RNAi-mediated knockdown of *TAR2* in the stellate cells eliminated the large majority of the TA response, the same knockdown had no effect on the overall level of *TAR2* expression in the tubule. One interpretation of this result is that TAR2 is expressed in another cell type within the MT: either in the principal cells or at high levels in a minor cell type, such as muscle or tracheal cells. However, because we measured the effect of RNAi on transcript abundance only and not on receptor protein levels, our negative result must be interpreted with caution. It is possible that a small reduction in TAR2 transcript abundance resulted in a large decrease in functional receptor expression in the stellate cells.

The TAR3 subtype of TA receptor is reported to have arisen recently in evolution, being only present in members of the *Drosophila* genus, and to have an expression pattern and agonist specificity distinct from that of *TAR2* ^29^. The relatively recent appearance of the TAR3 gene in insect evolution begs the question of whether this receptor subtype has a distinct physiological function. The reported ability of TAR3 to couple to both calcium release and inhibition of adenylyl cyclase, in contrast to TAR2^29^, suggests a possible function of this receptor in fine-tuning the response of the MT to TA. The new TAR mutant lines reported here should prove to be extremely useful in elucidating the function of the TAR2 and TAR3 receptors in many aspects of insect physiology and development.

## Methods

### Drosophila strains and maintenance

Stocks of *Drosophila melanogaster* were maintained on cornmeal/yeast/molasses/agar food at 24 °C on a 12 hr:12 hr light-dark cycle. The following stocks were used in these studies: *w; TAR3* ^*MB03028*^ (FBst0023837-BL), *w; TAR2* ^*MB09692*^ */TM3 Ser* (FBst0027797-BL), *y v; TAR2* ^*JF01878*^ (FBst0025857-BL), *w; TAR2* ^*f05682*^ */TM3 Ser* (FBst1020033-Exelixis), *y v; TAR3* ^*JF02749*^ (FBst0027670-BL), *w; P{GawB}c42* (FBst0030835, gift of Prof. Julian Dow), and *w; P{GawB}c710* (FBti0009567, gift of Prof. Julian Dow), w; *P{UAS-Dcr-2.D}10 P{GawB}c42 and* w; *P{UAS-Dcr-2.D}10 P{GawB}c710* 59*, P{FRT} PBac{DsRed}TAR2* ^*LL06812*^(*Drosophila Genetic Resource Center*)*, w* ^*1118*^ *; sna* ^*Sco*^ */SM6a, P{hsILMiT}2.4* (*FBst0024613-BL*). Genes studied in this work include *TAR2* (*TyrR*) (FBgn0038542)*, TAR3* (*TyrRII*) (FBgn0038541), and *RpL32* (*FBgn0002626*).

### Creation of recombinants and receptor mutants

Recombinants of *TAR2* ^*f05682*^ with the *c42* or *c710-gal4* drivers and the *TAR2* ^*JF01878*^ and *TAR3* ^*JF02749*^ RNAi transgenes were created by standard crosses. Transgenes were followed by eye color where possible and confirmed by PCR (see Table S1 for primer sequences). New TA receptor mutants were created with the following crossing scheme:

1. *w; noc* ^*Sco*^ */SM6a, hsILMiT x w; TM6b/*+ → *w; SM6a, hsILMiT/*+; *TM6b/*+
2. w; SM6a, hsILMiT/+; TM6b/+ x w; TAR3^MB03028^/TAR3^MB03028^ → *w;* SM6a, *hsILMiT/*+; *TAR3* ^*MB03028*^ */TM6b*
3. *w;* *SM6a, hsILMiT/*+; *TAR3* ^*MB03028*^ */TM6b x w* *;* *TAR2* ^*MB09692*^ */TAR2* ^*MB09692*^ → *w;* *SM6a, hsILMiT/*+; *TAR3* ^*MB03028*^ */TAR2* ^*MB09692*^
  Two days after setting up cross 3, flies were transferred to new vials and heat-shocked daily for 1 hr at 37 °C until pupariation to induce transposase expression.
4. *w;* *SM6a, hsILMiT/*+*; TAR3* ^*MB03028*^ */TAR2* ^*MB09692*^ *x w; TM3 Ser/*+ → *w; Df*?*/TM3 Ser*

Balanced lines were established from individual cross 4 progeny that lacked GFP expression and screened by PCR for deletion of the *TAR2* and *TAR3* genes (see Table S1).

### Quantitative RT-PCR

Posterior MTs were dissected from groups of 7–8 6 to 8 day old adult *Drosophila* females. For RNAi knockdowns, dissected flies were progeny of crosses between either *UAS-Dcr2 c42-gal4* or *UAS-Dcr2 c710-gal4* and either *y v; TAR2* ^*JF01878*^ or *y v; TAR3* ^*JF02749*^. Progeny from crosses in both directions were used (i.e. gal4 driver females x RNAi males, and gal4 driver males x RNAi females). MT RNA was isolated and DNAse treated (RNaqueous Micro kit, Thermo Fisher Scientific, Waltham, MA), and 50% of each RNA prep was then used as a template for cDNA synthesis (Qscript, Quansys Biosciences, Logan, UT). Quantitative PCR was performed using Perfecta SYBR mastermix (Quansys), 250 nM primers, 0.1 uL cDNA per reaction in a CFX thermocycler (Bio Rad, Hercules, CA) and analyzed using CFX Manager software (Bio Rad). Primer sequences are shown in Table S1. Each PCR run contained dilution series of one cDNA with all tested primer sets, and the resulting standard curves were fit with straight lines (r^2^ ≥ 0.98, efficiency 85–101%) and used to quantify unknowns. Levels of *TAR2* expression were normalized to those of the housekeeping gene *RpL32*.

### Electrophysiology

MTs were dissected from 6 to 8-day old adult *Drosophila* females and placed in dishes in which 100 μL of 31 μg/mL poly-L-lysine was allowed to dry and that were rinsed with deionized water briefly before dissection, and the TEP was recorded with a conventional microelectrode as described^60^. Data acquisition and analysis was performed with pClamp 9 software (Molecular Devices, Sunnyvale, CA). The dissecting and recording saline consisted of the following (in mM): 85 NaCl, 20 KCl, 3 CaCl_2_, 12 MgCl_2_, 7.5 NaHCO_3_, 10 HEPES, 15 glucose, pH 6.8 (260–265 mOsm). Agents added to the recording solution included TA (Sigma-Aldrich, St. Louis, MO), OA (Pfaltz & Bauer, Waterbury, CT), and drosokinin (AnaSpec, Fremont, CA). Recording saline osmolality was measured with a vapor-pressure osmometer (Wescor, Logan, UT).

Response index values for TA and drosokinin responses were calculated as previously described^60^.

### Statistics

For statistical analysis, response index values were transformed by taking the arcsine of the square root of each value. Negative values were transformed by taking the negative arcsine of the square root of the absolute value of the response index. Statistical tests were performed with Graphpad Prism v5.02 for Windows (GraphPad Software, San Diego, CA, www.graphpad.com). Datasets were tested for normality using a D’Agostino-Pearson normality test (this test is only valid for n ≥ 8). Datasets that failed this test were analyzed using non-parametric statistics; see figure legends for details. Error bars in figures indicate standard deviations.

## Electronic supplementary material

Supplemental Data
