# Supplementary material for: Identification of multiple functional receptors for tyramine on an insect secretory epithelium
Source: Sci Rep. 2017 Mar 13;7:168. doi: 10.1038/s41598-017-00120-z (PMC5427925; doi:10.1038/s41598-017-00120-z)
Supplement: Supplementary file 1 — Supplemental Data [file 41598_2017_120_MOESM1_ESM.pdf]

Identification of multiple functional receptors for tyramine on an insect secretory epithelium

Haiying Zhang and Edward M. Blumenthal*

Supplemental Data

Table S1: PCR primers

Primer pair	left primer	right primer
A	AAGATCGTGAAGAAGCGCACCAA	CTGTTGTCGATACCCTTGGGCTT
B	GTCTTTGCCACCTTCATCGT	TCCAGGGAGATCCAAATGTC
C	GATCATGATGAGGGCCAATC	CAGTGACACTTACCGCATTGACAAGCACGC
D	TAAAAAACCTCCCACACCTCCC	GAGCTCGTTGTTGGTTGGCA
E	GCATCGTCGGGCTGGCATAT	CAATCATATCGCTGTCTCACTCA
F	CAATCATATCGCTGTCTCACTCA	GAACAAAAGCGCCATTTCGG
G	CCATGGGCTCCTTCTTCATA	GGTCGTGTGAGTTCGTAGCA
H	GCAAAATATCTTGTGAGTGGTGTGCG	GTGCTGGTTAATTTGGGCGC
I	TGACATTCATCCGGGGTCAG	AAAATCTGATACGCAGCACG
J	TTTTGGCCTGGCCAGAAAGC	CGGACGTGGAGAATGAGCTG
K	TGGACATCCTGCTCTGCAGC	CCATGGGCTCCTTCTTCATA

The table lists the sequences for PCR primers used in this study. A: *RpL32* quantitative PCR. B: *TAR2* quantitative PCR. Note that the amplimer produced from this primer pair does not overlap with the ds-RNA produced from the $TAR2^{JF01878}$ RNAi transgene. C: confirming the $TAR2^{f05682}$ insertion. D: identifying the $TAR2^{JF01878}$ and $TAR3^{JF02749}$ transgenes in potential recombinants. E: identifying the *c42-gal4*

transgene in potential recombinants. F: identifying the *c710-gal4* transgene in potential recombinants. G and H: screening potential deletion lines for deletion of *TAR2* and *TAR3*, respectively. I, J, and K: confirmation of *TAR3*$^{\Delta 29}$, *Df(3R)TAR*Δ*30*, and *Df(3R)TAR*Δ*124* deletions, respectively.

Supplementary Figure S1

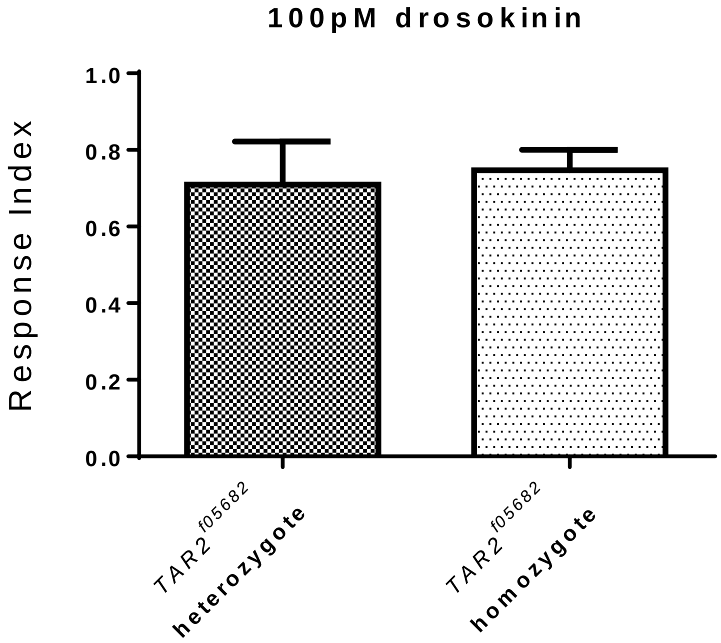

Figure S1. Mutation of TAR2 does not affect responses to drosokinin. Mean responses of tubules from *TAR2*^{f05682} heterozygotes and homozygotes to 100 pM drosokinin are shown. There was no difference between the two genotypes, p=0.45 Mann-Whitney test, n=6-7 tubules per genotype.

Supplementary Figure S2

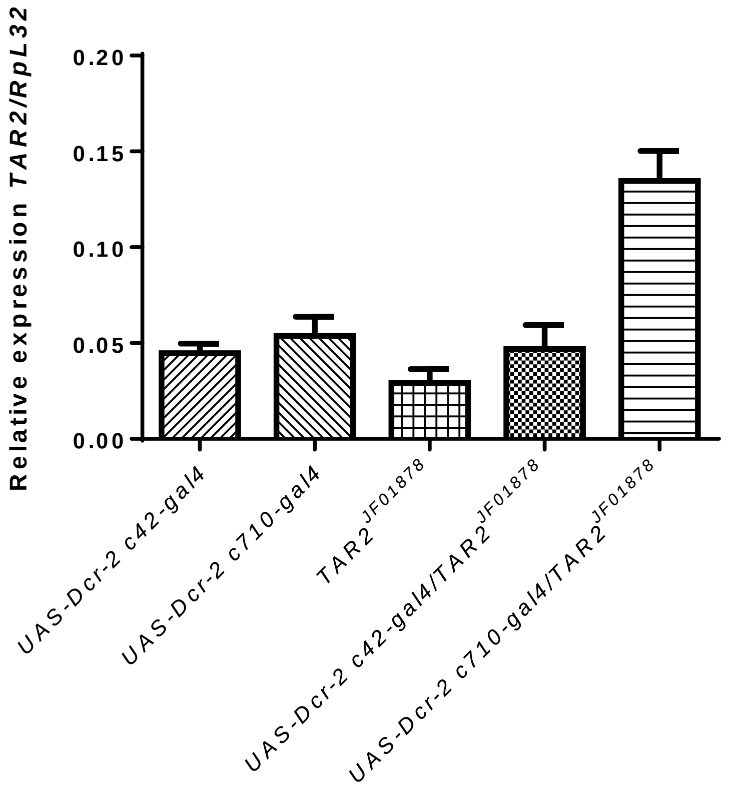

Figure S2. Inducing RNAi against TAR2 in either the principal or stellate cells does not result in a reduction in transcript abundance in the MT as a whole. Normalized TAR2 expression levels are shown for the three parental lines (first three bars), principal cell RNAi (fourth bar), and stellate cell RNAi (fifth bar). A Kruskal-Wallis and Dunn's multiple comparisons test showed a significant difference only between the RNAi parental line and stellate cell knockdown. N=3 independent cDNA preps per genotype.

Supplementary Figure S3

A

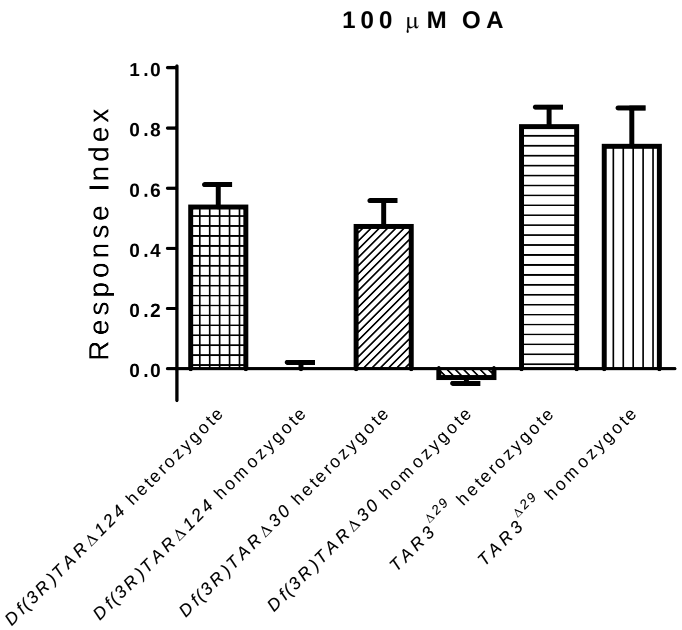

B

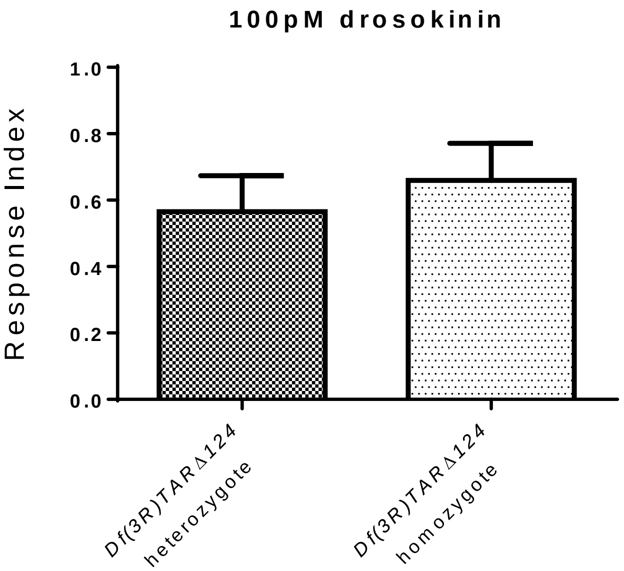

Figure S3. A: responses of deficiency mutations to 100μM OA. N=5-8 tubules per genotype. There was no difference in response amplitude between *TAR3*$^{\Delta 29}$ heterozygotes and homozygotes, p=0.22,

unpaired t-test. Average response amplitude in *Df(3R)TARΔ124* homozygotes did not differ from zero, p=0.97, one-sample t-test, while the average response of *Df(3R)TARΔ30* homozygotes was significantly less than zero, p=0.0002. B: responses of *Df(3R)TARΔ124* heterozygotes and homozygotes to 100pM drosokinin. N=9-10 tubules per genotype. No difference was seen between the genotypes, p=0.07 Mann-Whitney test (a non-parametric test was used because the homozygote dataset was not normally distributed).
